# Chromosomal assignment of centromere-specific histone CENH3 genes in rye (*Secale cereale* L.) and their phylogeny

**DOI:** 10.3897/CompCytogen.v11i4.19953

**Published:** 2017-12-14

**Authors:** Yulia A. Lipikhina, Elena V. Evtushenko, Evgeny A. Elisafenko, Alexander V. Vershinin

**Affiliations:** 1 Institute of Molecular and Cellular Biology SB RAS, Novosibirsk, 630090, Russia; 2 Institute of Cytology and Genetics SB RAS, Novosibirsk, 630090, Russia

**Keywords:** Centromeric histone CENH3, rye, wheat-rye addition lines, barley, Triticeae

## Abstract

Centromeres are essential for correct chromosome segregation during cell division and are determined by the presence of centromere-specific histone 3 (CENH3). Most of the diploid plant species, in which the structure and copy number of *CENH3* genes have been determined, have this gene as a singleton; however, some cereal species in the tribe Triticeae have been found to have CENH3 in two variants. In this work, using the set of the wheat-rye addition lines we wanted to establish the chromosomal assignment of the *CENH3* genes in the cultivated rye, *Secale cereale* (Linnaeus, 1753), in order to expand our knowledge about synteny conservation in the most important cereal species and about their chromosome evolution. To this end, we have also analyzed data in available genome sequencing databases. As a result, the αCENH3 and βCENH3 forms have been assigned to rye chromosomes 1R and 6R: specifically, the commonest variants αCENH3v1 and βCENH3v1 to chromosome 1R, and the rare variants, αCENH3v2 and probably βCENH3v2, to chromosome 6R. No other CENH3 variants have been found by analysis of the rye genome sequencing databases. Our chromosomal assignment of CENH3 in rye has been found to be the same as that in barley, suggesting that both main forms of CENH3 appeared in a Triticeae species before the barley and wheatrye lineages split.

## Introduction

In centromeric nucleosomes, canonical histone H3 appears in the form of its centromere-specific modification denoted in plants as CENH3. The presence of this protein is by far the most distinct molecular feature of this chromosomal region. Unlike canonical histone H3, which has a conserved structure, CENH3 normally shows considerable variability across species (Sanei et al. 2011, Maheshwari et al. 2015, Neumann et al. 2015). Different domains of this molecule are diverging differently. An extended Nterminal tail (NTT) domain and loop 1 of the histone fold domain (HFD) putatively interact with centromeric DNA (Vermaak et al. 2002) and show signatures of positive selection in some animal and plant species (Malik and Henikoff 2001, Talbert et al. 2002), while the part of the HFD that lies outside loop 1 is generally conserved (Talbert et al. 2002, Cooper and Henikoff 2004, Hirsch et al. 2009, Finseth et al. 2015).

Most of the diploid plant species, in which the structure and copy number of *CENH3* have been determined, have this gene as a singleton. Cereal species as these are, for example, maize and rice (Zhong et al. 2002, Nagaki et al. 2004), which are phylogenetically distant from the tribe Triticeae (the subfamily Pooideae, Dumort, 1824), which includes the closely related genera *Triticum* Linnaeus, 1753, *Secale* Linnaeus, 1753 and *Hordeum* Linnaeus, 1753. The closest relatives to rye in Triticeae, namely, tetraploid wheat species (Yuan et al. 2015), diploid barley, wheat and *Aegilops* Linnaeus, 1753 species (Sanei et al. 2011, Yuan et al. 2015), have been found to have *CENH3* in two variants. Thus, the presence of at least two copies of the *CENH3* gene is a shared feature of the species in the tribe Triticeae, and this gene had probably been duplicated before the barley and wheatrye lineages split, which is variously reported to date back to 89 MYA (Middleton et al. 2014) or 11.6 MYA (Martis et al. 2012). Using wheat-barley addition and substitution lines, the chromosomal assignment of the *CENH3* genes in two barley species, *H. vulgare* Linnaeus, 1753 and *H. bulbosum* Linnaeus, 1756, was established as encoding by chromosomes 1H and 6H (Sanei et al. 2011). In hexaploid wheat, *CENH3* genes were assigned to chromosome 1 in all the homeologous genomes, AA, BB and DD (Li et al. 2013, Yuan et al. 2015). However, so far the chromosomal localization of the *CENH3* genes in the other Triticeae species has been beyond the scope of any study known to us.

It has been established by comparative RFLP (restriction fragment length polymorphism) that the rye genome shares extensive synteny with the barley and wheat genomes (Devos et al. 1993). Accumulation of rye genome sequencing data (Martis et al. 2013, Bauer et al. 2017) enabled a genome-wide high-density comparative analysis at a new level and identified six major translocations that had shaped the modern rye genome and made it different from a putative Triticeae ancestral genome (Martis et al. 2013). In this work, we characterize the molecular structure of the main forms of CENH3 protein in rye, *Secale cereale*. Also, we attempt to assign the *CENH3* genes to particular chromosomes, using a set of seven wheat-rye addition lines, each containing a rye chromosome in the wheat genome. A comparison of the localization sites of so functionally important genes in closely related genera is expected to improve our knowledge about conservation of synteny between the most important cereal species. To this end, available databases with the DNA sequences of rye, *S. cereale*, and diploid species as donors of the hexaploid genome of *T. aestivum* Linnaeus, 1753 have been analyzed and the phylogenetic relationships between the different variants of *CENH3* have been inferred.

## Material and methods

### Plant material and plant growth

The plant material used were the bread wheat ‘Chinese Spring’ (CS) (2n=6x=42, AABBDD), the rye cultivar Imperial (2n=2x=14, RR) and wheat-rye (‘Chinese Spring’/’Imperial’) disomic addition lines involving rye chromosomes 1R–7R (Driscoll and Sears 1971). ‘Chinese Spring’ is an international standard for wheat research, much as ‘Imperial’ is for rye. Seeds were from the germplasm collection of the Leibniz Institute of Plant Genetics and Crop Plant Research (IPK), Gatersleben, Germany. All plants were grown in a greenhouse at 22 °C/18 °C (day/night) with a 16h day length.

### RNA isolation and PCR amplification

Total RNA was isolated from leaves of 12dayold seedlings using the TRI Reagent (MRC, Inc., USA) and treated by RQRNaseFree DNase (Promega, Madison, WI) according to the manufacturer’s instructions. RNA was reverse-transcribed to cDNA using a RevertAid H Minus First Strand cDNA Synthesis Kit (Thermo Fisher Scientific). The specific primers used to amplify the *CENH3* gene from cDNA were:

1) 5’ATGGCCCGCACCAAGCAC3’, 5’GCATCACCAAAGCCTCC3’, to amplify the coding region of αCENH3; and

2) 5’TGGGTCGCACGAAGCAC3’, 5’TCACCAAAGCCTTCTCCCC3’, to amplify the coding region of βCENH3.

### Cloning and sequencing

RTPCR products were purified using a Qiagen Purification Kit (Qiagen) and cloned using an InsTAclone PCR Cloning Kit (Thermo Fisher Scientific). Both strands of each of 15–20 clones of each parental variety and addition line were sequenced using an ABI 3130×1 Genetic Analyzer (Applied Biosystems Inc., CA) and an ABI BigDye Kit according to a standard protocol. Similarity searches between the *CENH3* sequences and their orthologs in other species were carried out using the TBLASTN software (Altschul et al. 1990) in the NCBI database (http://blast.ncbi.nlm.nih.gov/Database/).

### Sequence alignments and phylogenetic analysis

Amino acids alignments were performed online using Clustal Omega (Sievers et al. 2011) (http://www.ebi.ac.uk/Tools/msa/clustalo) and used for downstream analysis and visualization (Waterhouse et al. 2009) (http://www.jalview.org). The phylogenetic tree based on amino acid sequences was constructed using the Neighbor Joining algorithm in MEGA6 (Tamura et al. 2013). Bootstrap values were calculated from 1000 replicates.

### Analysis of databases

The search for rye genomic *CENH3* sequences was performed in among entries in the Sequence Read Archive (European Bioinformatics Institute, accession ID ERP001745) for sorted rye chromosomes 1R7R (Martis et al. 2013). The sequences of *CENH3* in the putative Agenome donor *Triticum urartu* Thumanjan ex Gandilyan, 1972 (accessions KM507181 and KM507184), the putative Bgenome donor *Aegilops speltoides* Tausch, 1837 (accessions KM507182 and KM507185), and the putative Dgenome donor *Aegilops tauschii* Cosson, 1849 (accessions KM507183 and KM507186) were downloaded from NCBI. Genomic DNA sequences were aligned with *CENH3*-coding sequences by the MartinezNW method using Lasergene’s MegAlign application.

## Results

### Assignment of the *CENH3* variants to rye chromosomes

**Figure 1.**
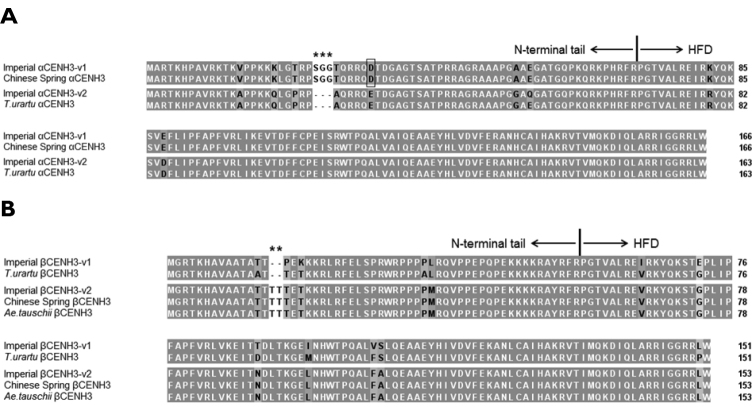
Examples of amino acid alignments of the different variants of the main forms of *CENH3* in rye *S. cereale* (cv. Imperial) and Triticeae species: *T. urartu* (KM507181, KM507184) and *Ae. tauschii* (KM507186). **A** αCENH3 **B** βCENH3. Asterisks are above positions with short insertions/deletions in the Nterminal tail; the position with the highest percentage of amino acid substitutions is framed.

We characterize two main forms of CENH3 proteins, αCENH3 and βCENH3, and their variants, according to differences in size and amino acid substitutions. The α*CENH3v1* cDNA sequence in the cultivated rye *S. cereale* is 501 bp in length and the associated protein consists of 166 amino acids. In *S. cereale*, β*CENH3v1* is distinct from α*CENH3v1* in that the former has several deletions in the NTT and the insertion of three nucleotides, ACC, which encode the amino acid threonine, in the HFD. Thus, β*CENH3v1* has an overall length of 456 bp and encodes a protein made up by 151 amino acids. Most of the NTT amino acid sequences in αCENH3 and βCENH3 do not align well with each other, the alpha and beta forms share as low as 58% nucleotide identity of the first 60 nucleotides from the 5’end. In addition to these main forms, their much less common variants (minor, throughout) were found. The α*CENH3v2* sequences were 492 bp in length each, that is, they were shorter α*CENH3v1*. Additionally, these two αCENH3 variants have different amino acids at some positions. Some rye accessions carry *CENH3* sequences that are individually 6 bp longer than β*CENH3v1* and encode two additional amino acid residues of threonine in the NTT domain (Fig. 1B). We classify sequences as these as βCENH3v2. The two βCENH3 variants have different amino acids at some positions, too.

The amino acid differences between CENH3 in rye and wheat were used for the chromosomal assignment of the *CENH3* copies in *S. cereale*. Each of the seven wheat-rye addition lines ‘Chinese Spring’/‘Imperial’ (2n=44 (42+2R)) (Driscoll and Sears 1971) contains the entire hexaploid wheat *Triticum aestivum* genome from ‘Chinese Spring’ and a pair of rye *S. cereale* chromosomes from ‘Imperial’. A comparison of the sequences of the alpha variants of *CENH3* cDNA between ‘Imperial’ and ‘Chinese Spring’ revealed a high (99%) level of identity and it is no wonder why the consensus amino acid sequences of ‘Imperial’ αCENH3v1 and ‘Chinese Spring’ αCENH3 were found to be identical (Fig. 1A). The 100% amino acid sequence identity between rye and wheat prevents this variant from being assigned to particular rye chromosomes using addition lines. Several positions in these sequences are polymorphic. The most frequently observed polymorphism is at position 33 of the NTT domain (framed in Fig. 1A), where glutamine acid instead of asparagine acid is present in 20% of the ‘Imperial’ clones and in 47% of the ‘Chinese Spring’ clones.

**Figure 2.**
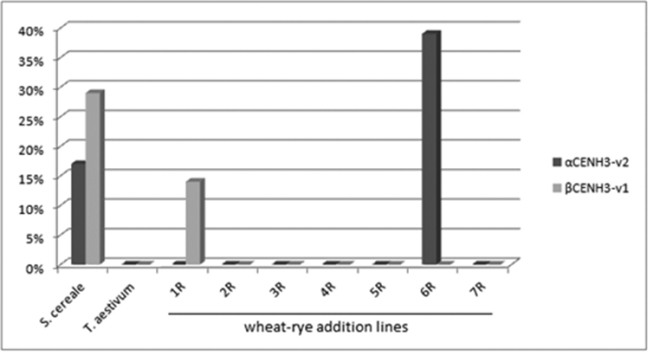
Histogram showing the percentage of α*CENH3*-v2 and β*CENH3*-v1 in the cDNA clones from rye *S. cereale* (cv. Imperial), wheat *T. aestivum* (cv. Chinese Spring) and wheat-rye (1R–7R) addition lines.

Sixteen percent of the cDNA clones of the alpha variants of ‘Imperial’ *CENH3* have a 9 bp deletion and represent the minor variant, α*CENH3v2*, according to our classification (Fig. 1A). None of the 19 ‘Chinese Spring’ clones was found to have this deletion, allowing us to use addition lines for assigning this variant of *CENH3* to rye chromosomes. There was only one addition line (that with rye chromosome 6R), whose clones (39%) had this deletion (Fig. 2). None of the other addition lines had any clone with this deletion.

The shorter, 456bp-long forms of ‘Imperial’ *CENH3* DNA produce protein molecules, each containing 156 amino acids and collectively denoted as βCENH3v1 (Fig. 1B). They are less common than α*CENH3* and make up 29% of the clones. These sequences did not occur from ‘Chinese Spring’ DNA amplification and thus their chromosomal assignment using addition lines was possible. There was only one addition line (that with rye chromosome 1R), whose clones (specifically, 14%) were found to have sequences with this structure and of this size (Fig. 2). ‘Chinese Spring’ cDNA became the source of *CENH3* sequences, each containing a 6bp insertion in the NTT domain and corresponding, according to our classification, to β*CENH3*v2 in ‘Imperial’ DNA. The nucleotide sequence homology of this variant between wheat and rye is 100% and thus addition lines were of as little help in assigning this variant to particular rye as they were with α*CENH3v1*.

### Analysis of databases and phylogenetic relationships between *CENH3* in rye and wheats

To confirm the chromosomal assignment of various *CENH3* variants made using addition lines and to assign α*CENH3*v1 and β*CENH3*v2 to particular chromosomes, we analyzed entries in the Sequence Read Archive (European Bioinformatics Institute, accession IDERP001745) for sorted rye chromosomes 1R7R (Martis et al. 2013). Some sequence reads from chromosome 1R (accession ERX140512) were found to have a high nucleotide identity, 87–97%, of the coding sequence of α*CENH3v1*. That the contig composed of these reads was really α*CENH3v1* was indicated by the absence of the 9bp deletion, typical of α*CENH3v2*, in one of the reads (ERX140512.2250257), which contained the first exon of α*CENH3* entirely and the coding amino acid sequence identical to αCENH3v1.

Additionally, two of the reads from chromosome 1R were found to contain the coding region (positions 1 through 328) of β*CENH3v1* (ERX140512.1955393, ERX140512.290111): they had no 6bp insertion in the NTT domain that all β*CENH3v2* normally have and they had large deletions in the NTT that delineate beta forms from alpha forms. Some sequence reads from chromosome 6R (accession ERX140517) were found to have a high nucleotide identity to the HFD in β*CENH3*. Because these reads contained only the most conserved region of the HFD (the last 42 amino acids) and because this region was identical between β*CENH3v1* and β*CENH3v2*, we were unable to tell these variants from each other, however, one thing was clear: β*CENH3* is located on chromosome 6R. Thus, the analysis of the Sequence Read Archive for gDNA sequences amplified from sorted rye chromosomes 1R7R assigned α*CENH3v1* to chromosome 1R, the beta form of *CENH3* to chromosome 6R and confirmed the addition line-based assignment of β*CENH3v1* to chromosome 1R. In summary, the main forms of *CENH3* (the alpha and beta forms) are located on rye chromosomes 1R and 6R, the commonest variants, α*CENH3v1* and β*CENH3v1*, are on chromosome 1R, and the less common α*CENH3v2* and probably β*CENH3v2* are on chromosome 6R. It should be noted that analysis of the most recent version of the rye genome without chromosome sorting (Bauer et al. 2017) did not reveal any *CENH3* variants other than those described herein.

**Figure 3.**
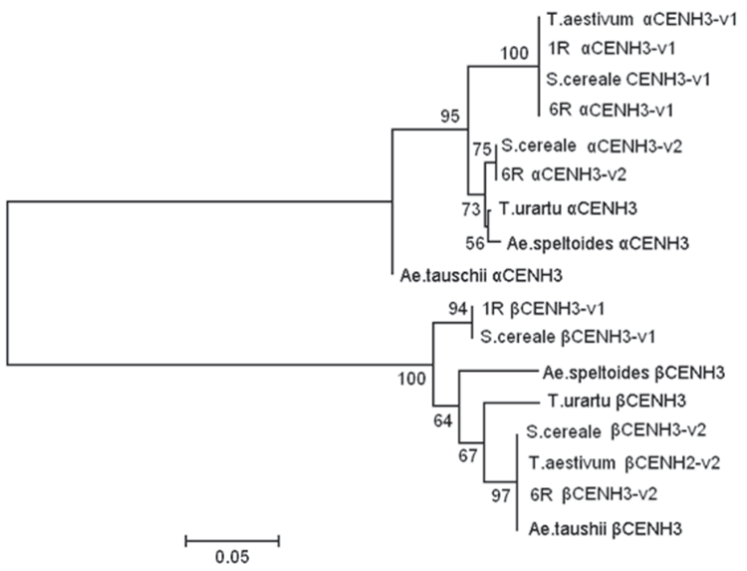
Phylogenetic tree of the CENH3 proteins. Phylogenetic tree inferred using JTT+G models (measures distances) and bootstrapping (1000 replicates). Bootstrap values are indicated on the branches. Rye *S. cereale* (cv. Imperial), wheat *T. aestivum* (cv. Chinese Spring) and wheat-rye (1R–7R) addition lines. NCBI accessions are: αCENH3 and βCENH3 in *T. urartu* (KM507181, KM507184), *Ae. tauschii* (KM507183, KM507186), *Ae. speltoides* (KM507182, KM507185). The scale bar is substitutions per site.

A high level of identity of *CENH3* sequences between wheat and rye is not consistent with a wealth of plant species data that suggest considerable between-species differences in the structure of this protein (Sanei et al. 2011, Masonbrink et al. 2014, Dunemann et al. 2014, Maheshwari et al. 2015). This encouraged us to explore the phylogenetic relationships between the different *CENH3* variants in rye, wheat and diploid species seen as putative donors of the hexaploid wheat genomes. As was found out, *T. urartu* accession KM507181 shares a high (99%) nucleotide identity with ‘Imperial’ α*CENH3v2*, and *A. tauschii* accession KM507186, 100% identity with ‘Imperial’ β*CENH3v2* (Fig. 1A, B). The neighborjoining phylogenetic tree for CENH3 amino acid sequences consists of two major clusters, one with alpha forms and another with beta forms (Fig. 3). The topology of the clusters displays some differences in the ways they branch. The bootstrap values in the beta cluster suggest its clearer partitioning into subclusters. In the alpha cluster, CENH3’s from *T. urartu* and *A. speltoides* share the same subcluster, while in the beta cluster, each of these species forms a separate branch. CENH3 sequences obtained from the lines, in which added chromosomes (particularly, 1R and 6R) were found to carry rye *CENH3* copies, share the same subcluster with the ‘Imperial’ sequences. The major variants (variants 1) form prominent subclusters, while the minor variants (variants 2) lie closer to the diploid species *T. urartu, A. tauschii* and *A. speltoides*. Although these species are considered the main contributors to the hexaploid wheat genome, the level of CENH3 identity between them and the rye cultivar Imperial is just as high.

## Discussion

Most of the 67 plant species with the CENH3 sequence publicly available – including those that have undergone whole-genome duplication – have this essential gene as a singleton (Maheshwari et al. 2015). Note that sampling bias is a factor here, for 23 species (1/3) were from Brassicaceae. In cereals, three CENH3 variants described in the oats *Avena sativa* Linnaeus, 1753 (Ishii et al. 2015) represent the alpha form and two forms of CENH3 appear for the first time in the closest rye relatives: the Triticeae genera barley, wheat and *Aegilops* (Sanei et al. 2011, Yuan et al. 2015). Comparative analyses of the cereal genomes revealed a whole-genome duplication (WGD), which occurred between 53 and 94 MYA (i.e. before the divergence of the cereal genomes), a recent segmental duplication between chromosomes 11 and 12 and numerous individual gene duplications (Feuillet and Salse 2009). Because the Triticeae species carry a haploid set of seven chromosomes, it is hypothesized that the two forms of CENH3 emerged from a duplication event (Yuan et al. 2015), which in this case is most likely to represent a local duplication event that had taken place before the barley and wheat-rye lineages split.

The now commonly accepted viewpoint authored by Ohno (1970) is that a local duplication event gives rise to two functionally redundant, paralogous genes, one of which will carry on under attenuated selective pressure and will thus be freed from selective constraints. As a result, each copy as this starts to accumulate mutations that would have been deleterious to the gene, if it were a singleton. In reference to *CENH3* in rye, wheat and barley, it can be hypothesized that the copies accumulating deleterious mutations are the beta forms, because their NTT domains contain several deletions of various size that the NTT domains of the alpha forms do not. Interestingly, inactivation of beta CENH3 in barley did not result in a major phenotype and point mutation impairs centromeric *CENH3* loading and induces haploid plants (Karimi-Ashtiyani et al. 2015). The structure of *CENH3* in cereal species that have this gene as a singleton (for example, the rice *O. sativa*) is much closer to the structure of the Triticeae alpha forms (74% identity, while no alignment with the beta form is possible because of extended deletions), which can be regarded as being evolutionarily more ancient than beta forms. According to the theory of evolution by gene duplication (Ohno 1970, Wagner 2002), deleterious mutations should accumulate and lead to a rapid loss of one of the paralogs; however, this is not the case with *CENH3* copies in Triticeae. Among the possible reasons for the observed inconsistency with the theory is the acquisition of new functions by a duplicate gene or by both copies or sub- or neofunctionalization (Conant and Wolfe 2008). For example, in wild tetraploids of wheat, β*CENH3* has a much lower expression level than α*CENH3*, while in cultivated tetraploids β*CENH3* transcripts are enhanced to near α*CENH3* level (Yuan et al. 2015). All the *CENH3* variants that we have described have been found in rye cDNA, that is, they are transcribed; however, to understand the function of each particular variant, special research is required.

According to our results, both main forms, α*CENH3v1* and β*CENH3v1*, are located on rye chromosome 1R. Obviously, these copies do not reside there next to each other, but are somewhat spaced. There are two facts that support this statement. First, the two-copy organization of *CENH3* makes these copies very likely to end up with gene conversion events that homogenize their sequences (Wagner 2002). The presence of a large deletion in β*CENH3* and the heterogeneity of the nucleotide and amino acid sequences of the alpha and beta forms suggest quite the opposite. Secondly, analyzing *T. urartu* genome sequencing data (Ling et al. 2013), we found that α*CENH3* and β*CENH3* are 24 kb apart (locus KD187944.1, unplaced genomic scaffold 30245, BioProject PRJNA182347). Considering a high level of identity of *CENH3* sequences between rye and *T. urartu*, it is possible that the genomic region containing *CENH3* has remained highly syntenic between the rye and wheat genomes.

Various molecular mechanisms have been proposed to explain the emergence of these spaced gene copies on the same or different chromosomes (Glover et al. 2015). Some of these mechanisms are 1) retroposition where genes are reverse transcribed and reinserted back into the genome; 2) ectopic recombination during double-strand break (DSB) repair; 3) transposable element mediated gene captured and moved throughout plant genomes; 4) exon shuffling. All these processes involve rearrangements in gene structure. However, the amount of data available so far is insufficient for making an unbiased judgement as to which of these mechanisms should be accepted.

A comparison of the genetic maps between rye and barley shows that rye chromosome 1R and barley chromosome 1H are fully collinear (Martis et al. 2013). Noteworthy, 1R is the only rye chromosome in which no large translocations have been found as the rye karyotype was being shaped from a Triticeae progenitor (Martis et al. 2013). It has previously been established that *CENH3* is located on barley chromosome 1H (Sanei et al. 2011). In wheat, three highly homologous *CENH3* genes have been assigned to chromosomes 1A, 1B and 1D by PCR amplification in nullisomic-tetrasomic lines (Li et al. 2013) and fluorescent in situ hybridization (Yuan et al. 2015). Thus, the presence of *CENH3* genes on chromosome 1 is a shared feature of Triticeae genera. The barley species *H. vulgare* and *H. bulbosum* have additionally been found to have β*CENH3* on chromosome 6H (Sanei et al. 2011). Taking together the existing data and our assignment of *CENH3* copies to rye chromosome 6R, it can be stated that, the *CENH3* genes appeared on chromosome 6 as they did on chromosome 1 in a Triticeae species before the barley and wheat-rye lineages split.
